# Supplementary material for: Time-series transcriptome analysis identified differentially expressed genes in broiler chicken infected with mixed Eimeria species
Source: Front Genet. 2022 Aug 8;13:886781. doi: 10.3389/fgene.2022.886781 (PMC9393255; doi:10.3389/fgene.2022.886781)
Supplement: Supplementary file 2 [file DataSheet1.ZIP › 7dpi_GO.Gsea.1625106495923/GOCC_ORGANELLAR_RIBOSOME.html]

Details for gene set GOCC\_ORGANELLAR\_RIBOSOME[GSEA]

|  || Dataset | TMM\_7dpi\_gct\_format\_7dpi\_gct\_format.Class\_4dpi.cls #PC\_versus\_NC.Class\_4dpi.cls #PC\_versus\_NC\_repos |
| Phenotype | Class\_4dpi.cls#PC\_versus\_NC\_repos |
| Upregulated in class | 1 |
| GeneSet | GOCC\_ORGANELLAR\_RIBOSOME |
| Enrichment Score (ES) | 0.6555324 |
| Normalized Enrichment Score (NES) | 3.1870115 |
| Nominal p-value | 0.0 |
| FDR q-value | 0.0 |
| FWER p-Value | 0.0 |
Table: GSEA Results Summary

  

Fig 1: Enrichment plot: GOCC\_ORGANELLAR\_RIBOSOME      
 Profile of the Running ES Score & Positions of GeneSet Members on the Rank Ordered List

  

| SYMBOL | TITLE | RANK IN GENE LIST | RANK METRIC SCORE | RUNNING ES | CORE ENRICHMENT || 1 | MRPS12 | na | 242 | 0.999 | 0.0153 | Yes |
| 2 | MRPS6 | na | 315 | 0.928 | 0.0423 | Yes |
| 3 | MRPL30 | na | 351 | 0.883 | 0.0708 | Yes |
| 4 | NDUFAB1 | na | 356 | 0.876 | 0.1017 | Yes |
| 5 | NDUFA7 | na | 371 | 0.857 | 0.1310 | Yes |
| 6 | MRPL43 | na | 504 | 0.741 | 0.1463 | Yes |
| 7 | MRPL23 | na | 564 | 0.704 | 0.1665 | Yes |
| 8 | MRPL12 | na | 579 | 0.694 | 0.1900 | Yes |
| 9 | MRPL57 | na | 643 | 0.665 | 0.2084 | Yes |
| 10 | MPV17L2 | na | 697 | 0.638 | 0.2267 | Yes |
| 11 | MRPL58 | na | 755 | 0.609 | 0.2436 | Yes |
| 12 | MRPL54 | na | 779 | 0.596 | 0.2629 | Yes |
| 13 | MRPS33 | na | 798 | 0.591 | 0.2824 | Yes |
| 14 | MRPL35 | na | 853 | 0.571 | 0.2983 | Yes |
| 15 | MRPL10 | na | 872 | 0.563 | 0.3168 | Yes |
| 16 | MRPL55 | na | 880 | 0.561 | 0.3362 | Yes |
| 17 | MRPS16 | na | 950 | 0.538 | 0.3496 | Yes |
| 18 | MRPL21 | na | 1141 | 0.472 | 0.3504 | Yes |
| 19 | MRPL15 | na | 1217 | 0.450 | 0.3602 | Yes |
| 20 | MRPS10 | na | 1264 | 0.437 | 0.3719 | Yes |
| 21 | MRPS28 | na | 1280 | 0.432 | 0.3860 | Yes |
| 22 | MRPL40 | na | 1282 | 0.431 | 0.4013 | Yes |
| 23 | MRPL45 | na | 1312 | 0.427 | 0.4141 | Yes |
| 24 | MRPL46 | na | 1380 | 0.409 | 0.4231 | Yes |
| 25 | MRPL28 | na | 1382 | 0.409 | 0.4375 | Yes |
| 26 | NSUN4 | na | 1403 | 0.404 | 0.4502 | Yes |
| 27 | MRPL9 | na | 1437 | 0.396 | 0.4616 | Yes |
| 28 | MRPL3 | na | 1475 | 0.387 | 0.4723 | Yes |
| 29 | MRPL42 | na | 1521 | 0.375 | 0.4818 | Yes |
| 30 | MRPL19 | na | 1535 | 0.370 | 0.4939 | Yes |
| 31 | MRPL47 | na | 1550 | 0.367 | 0.5058 | Yes |
| 32 | MRPS17 | na | 1651 | 0.345 | 0.5097 | Yes |
| 33 | MRPL34 | na | 1672 | 0.339 | 0.5201 | Yes |
| 34 | MRPS35 | na | 1686 | 0.336 | 0.5310 | Yes |
| 35 | MRPS11 | na | 1692 | 0.335 | 0.5425 | Yes |
| 36 | MRPL33 | na | 1696 | 0.333 | 0.5541 | Yes |
| 37 | MRPS18C | na | 1729 | 0.325 | 0.5630 | Yes |
| 38 | MRPL37 | na | 1736 | 0.324 | 0.5740 | Yes |
| 39 | CHCHD1 | na | 1745 | 0.321 | 0.5848 | Yes |
| 40 | MRPL44 | na | 1799 | 0.307 | 0.5912 | Yes |
| 41 | MRPL2 | na | 1843 | 0.299 | 0.5983 | Yes |
| 42 | MRPS21 | na | 1864 | 0.296 | 0.6071 | Yes |
| 43 | MRPL16 | na | 1884 | 0.291 | 0.6159 | Yes |
| 44 | MRPL53 | na | 1984 | 0.273 | 0.6173 | Yes |
| 45 | MRPL41 | na | 2082 | 0.257 | 0.6184 | Yes |
| 46 | MRPL24 | na | 2093 | 0.254 | 0.6266 | Yes |
| 47 | MRPL13 | na | 2109 | 0.251 | 0.6343 | Yes |
| 48 | MRPS9 | na | 2193 | 0.238 | 0.6358 | Yes |
| 49 | MRPL48 | na | 2228 | 0.232 | 0.6412 | Yes |
| 50 | MRPL14 | na | 2284 | 0.224 | 0.6446 | Yes |
| 51 | MRPS14 | na | 2305 | 0.222 | 0.6508 | Yes |
| 52 | MRPS18A | na | 2363 | 0.213 | 0.6536 | Yes |
| 53 | MRPS34 | na | 2496 | 0.193 | 0.6494 | Yes |
| 54 | MRPL18 | na | 2512 | 0.189 | 0.6549 | Yes |
| 55 | MRPS5 | na | 2649 | 0.170 | 0.6496 | Yes |
| 56 | MRPL39 | na | 2682 | 0.166 | 0.6528 | Yes |
| 57 | MRPL22 | na | 2747 | 0.158 | 0.6530 | Yes |
| 58 | MRPL20 | na | 2783 | 0.153 | 0.6555 | Yes |
| 59 | MRPL38 | na | 2918 | 0.137 | 0.6492 | No |
| 60 | DAP3 | na | 3039 | 0.121 | 0.6434 | No |
| 61 | MRPL27 | na | 3152 | 0.107 | 0.6378 | No |
| 62 | MRPS7 | na | 3210 | 0.100 | 0.6366 | No |
| 63 | MRPL51 | na | 3225 | 0.098 | 0.6390 | No |
| 64 | MRPS22 | na | 3240 | 0.095 | 0.6412 | No |
| 65 | MRPS31 | na | 3424 | 0.071 | 0.6284 | No |
| 66 | MRPS26 | na | 3453 | 0.068 | 0.6284 | No |
| 67 | MRPL32 | na | 3822 | 0.025 | 0.5985 | No |
| 68 | MRPS27 | na | 3855 | 0.021 | 0.5966 | No |
| 69 | MRPS2 | na | 4677 | -0.068 | 0.5302 | No |
| 70 | MALSU1 | na | 4809 | -0.082 | 0.5221 | No |
| 71 | MTG2 | na | 4930 | -0.096 | 0.5155 | No |
| 72 | MRPS36 | na | 5609 | -0.160 | 0.4644 | No |
| 73 | MRPL17 | na | 5768 | -0.177 | 0.4574 | No |
| 74 | MRPL50 | na | 6658 | -0.271 | 0.3926 | No |
| 75 | MTG1 | na | 7184 | -0.328 | 0.3603 | No |
| 76 | MRPS30 | na | 8183 | -0.444 | 0.2924 | No |
| 77 | NSUN3 | na | 10405 | -0.791 | 0.1345 | No |
Table: GSEA details [plain text format]

  

Fig 2: GOCC\_ORGANELLAR\_RIBOSOME      
 Blue-Pink O' Gram in the Space of the Analyzed GeneSet

  

Fig 3: GOCC\_ORGANELLAR\_RIBOSOME: Random ES distribution      
 Gene set null distribution of ES for **GOCC\_ORGANELLAR\_RIBOSOME**
